# Intranasal esketamine in treatment-resistant depression: Descriptive study of our clinical experience

**DOI:** 10.1192/j.eurpsy.2025.1297

**Published:** 2025-08-26

**Authors:** E. Abalde García, E. Lanchares Balboa, L. C. Ortega Sandoval, J. A. Campos Pérez, J. M. Olivares Diez

**Affiliations:** 1 Hospital Álvaro Cunqueiro, Vigo, Spain

## Abstract

**Introduction:**

Major Depressive Disorder (MDD) represents a significant challenge for global public health, profoundly affecting social and economic well-being. It is estimated that around 30% of patients do not respond to standard therapies, being classified as Treatment-Resistant Depression (TRD) patients. Intranasal ketamine emerges as a promising therapeutic option for these patients.

**Objectives:**

The objective of this study is to analyze the actual clinical use of intranasal esketamine in a sample of patients with treatment-resistant depression (TRD).

**Methods:**

A descriptive study of different socio-demographic and clinical variables was conducted to analyze the profile of TRD patients treated with intranasal ketamine in the Vigo Health Area.

**Results:**

A study was conducted on 33 TRD patients (48% women), with an average age of 52.77 years. In the current episode, it was observed that 24.2% had received electroconvulsive therapy and 41.7% had experienced at least one psychiatric admission. Additionally, 66.7% had visited the emergency room for psychiatric reasons, while 50% presented with comorbid anxiety, 15% with obsessive-compulsive personality disorder, and 21% with fibromyalgia.

The analysis showed that 88% of the participants had failed at least six lines of treatment, with an average of 4.18 concomitant treatments at baseline. The response to intranasal ketamine was evidenced, for the most part, in the second week of treatment, with a notable increase in the use of the 84 mg dose. In 60% of patients, the induction period was set at 4 weeks and a decrease in MADRS scale scores was observed at weeks 4, 8, and 12. No adverse effects were reported that had not been previously recognized in clinical trials. Adverse effects were rare and mild.

**Conclusions:**

The study indicates that patients presented with highly complex depression, which explains the prolonged induction periods. The good tolerability of ketamine has facilitated a rapid increase to 84 mg dosage in most cases. Although this sample does not align completely with the typical profile of patients who respond well, intranasal ketamine was effective in 70% of cases, similar to the findings of clinical trials. It is suggested that the accumulated knowledge about the drug has contributed to these clinical improvements. Additionally, a more flexible approach has helped patients with low expectations of response.

**Disclosure of Interest:**

None Declared

